# Validation of molecular markers associated with boron tolerance, powdery mildew resistance and salinity tolerance in field peas

**DOI:** 10.3389/fpls.2015.00917

**Published:** 2015-10-27

**Authors:** Muhammad Javid, Garry M. Rosewarne, Shimna Sudheesh, Pragya Kant, Antonio Leonforte, Maria Lombardi, Peter R. Kennedy, Noel O. I. Cogan, Anthony T. Slater, Sukhjiwan Kaur

**Affiliations:** ^1^Grains Innovation Park, Department of Economic Development, Jobs, Transport and ResourcesHorsham, VIC, Australia; ^2^AgriBio, Centre for AgriBioscience, Department of Economic Development, Jobs, Transport and ResourcesBundoora, VIC, Australia

**Keywords:** diagnostic marker, linked markers, QTLs, breeding, quantitative traits

## Abstract

Field pea (*Pisum sativum* L.) is an important grain legume consumed both as human food and animal feed. However, productivity in low rainfall regions can be significantly reduced by inferior soils containing high levels of boron and/or salinity. Furthermore, powdery mildew (PM) (*Erysiphe pisi*) disease also causes significant yield loss in warmer regions. Breeding for tolerance to these abiotic and biotic stresses are major aims for pea breeding programs and the application of molecular markers for these traits could greatly assist in developing improved germplasm at a faster rate. The current study reports the evaluation of a near diagnostic marker, PsMlo, associated with PM resistance and boron (B) tolerance as well as linked markers associated with salinity tolerance across a diverse set of pea germplasm. The PsMlo1 marker predicted the PM and B phenotypic responses with high levels of accuracy (>80%) across a wide range of field pea genotypes, hence offers the potential to be widely adapted in pea breeding programs. In contrast, linked markers for salinity tolerance were population specific; therefore, application of these markers would be suitable to relevant crosses within the program. Our results also suggest that there are possible new sources of salt tolerance present in field pea germplasm that could be further exploited.

## Introduction

Field pea (*Pisum sativum* L.) is an important grain legume consumed as an inexpensive source of proteins, complex carbohydrates, fiber, B group vitamins, folate and minerals such as calcium, iron and potassium. Peas contain up to 87% total digestible nutrients, which along with their low sodium and fat content (Tiwari and Singh, 2012) make them a valuable food source. In Australia, field peas are grown on over 280,000 ha per annum (ABARES, 2014) and over 70% of this production is within the lower rainfall (≤230 mm) cropping regions of southern Australia (Leonforte, 2013).

Soils in these low rainfall regions are often sodic, saline, and alkaline, and can have toxic levels of boron (B) (Rengasamy, 2006; Nuttall et al., 2010; Javid et al., 2014) that can significantly limit field pea growth and yield. Amelioration of these subsoil constraints is considered impractical from an economic or logistical perspective (Nuttall et al., 2010). Consequently, identification of germplasm that has a combination of B and salinity tolerance is likely to result in improved growth and yield in the low rainfall areas with harsh soils.

Boron concentrations in the range of only 10–54 mg kg^-1^ in the soil will inhibit plant growth (Reid et al., 2004), and soils in the southern Australian cropping region can range from 2 to 52 mg kg^-1^ B (Nuttall et al., 2003). The physiological mechanisms of B tolerance include reduced uptake into roots and reduced translocation into shoots, resulting in less accumulation of B in plant tissues (Reid and Fitzpatrick, 2009). Bagheri et al. (1996) identified two additive loci with incomplete dominance that conferred tolerance to high levels of B in peas. While tolerance controlled by single genes has been identified in the model legume *Medicago truncatula* (Bogacki et al., 2013) and lentil (Kaur et al., 2013). QTL mapping has also recently identified a single locus for B tolerance in field peas and tightly linked markers have been identified (Sudheesh et al., 2014).

Powdery mildew in peas is caused by *Erysiphe pisi* DC, and is a serious disease that can cause yield losses of 25–50% (Warkentin et al., 1996; Tiwari et al., 1997; Fondevilla and Rubiales, 2012). In Australia, the disease occurs frequently in parts of South Australia, Victoria, and New South Wales mainly in wetter regions, although it also occasionally occurs in drier shorter season pea growing areas (Davidson et al., 2004). The obligate biotrophic fungus flourishes with dewy nights and warm days, with an optimal temperature of 20°C for conidial germination (Davidson et al., 2004). The disease is most destructive in late-sown field pea crops and in late maturing cultivars (Davidson et al., 2004). Genetic resistance is important as it is more cost-effective and environmentally friendly than fungicide applications. The genetics of PM resistance in field pea is relatively well understood with three major loci (*er1, er2*, and *Er3*) reported (Fondevilla et al., 2008; Srivastava et al., 2012; Pavan et al., 2013). The *er1* locus is on chromosome Ps VI (Timmerman et al., 1994) and is tightly linked to B tolerance (Sudheesh et al., 2014). The *er1* resistance is considered stable and more effective than *er2* or *Er3.* The recessive *er1* locus is due to a loss-of-function mutation of *PsMlo1* (Mildew Resistance Locus O; Humphry et al., 2011).

Salinity is a persistent problem to crop productivity in low rainfall regions of the world (Rengasamy et al., 2003; Javid et al., 2012). Toxic levels of sodium in the subsoil, often associated with highly alkaline soils, are the main cause of yield loss associated with salinity (Rengasamy, 2002, 2006). Salinity negatively affects nearly all developmental stages in plants (Flowers et al., 2010), and these effects are generally due to water deficit, ion toxicity and ion imbalance (Marschner, 1995). Munns and Tester (2008) reported that plant growth responds to salinity in two phases; a rapid osmotic phase, and a slower ionic phase. Whole plant tolerance to salinity is achieved through osmotic adjustment, ion exclusion (avoidance) and compartmentalization of sodium and chloride ions (Blumwald, 2000; Munns and Tester, 2008). In recent years, salinity tolerance in field pea has become increasingly important in Australia due to a geographical shift of crop production toward environments characterized by shorter seasons, reduced water availability and marginal soils with higher transient soil salinity (Nuttall et al., 2010; Leonforte et al., 2013b). The genetics of plant salinity tolerance is complex and governed by multiple genes with small effects (Arraouadi et al., 2012; Leonforte et al., 2013b), and is highly affected by environmental conditions. However, with molecular markers, the genetic analysis of quantitative inheritance of salinity tolerance is possible (Koyama et al., 2001), and Leonforte et al. (2013b) have identified markers for two salinity QTLs [Vp = 12% (QTL-1), 19% (QTL-2)] from the moderately tolerant field pea cultivar Parafield.

The application of molecular markers has the ability to enable breeders to select germplasm and breeding lines on the basis of a simple cost-effective DNA assay without the need to undertake extensive phenotypic evaluation. These markers must be reliable and closely linked to reduce the probability of recombination. The procedure must be straight forward so that it can be done in a timely manner within a breeding program and it must be efficient to enable significant cost and time savings (Collard et al., 2005; Collard and Mackill, 2008). These savings were estimated to be 40% in wheat (Kuchel et al., 2005), and 75% in common bean (Yu et al., 2000) and potato (Slater et al., 2013).

The main objective of this work was to assess a near diagnostic marker PsMlo1 (Sudheesh et al., 2014) for B tolerance and PM disease resistance, and tightly linked markers for two salinity tolerance QTLs (Leonforte et al., 2013b) in a diverse set of field pea genotypes for potential application in pea breeding programs.

## Materials and Methods

### Phenotyping

#### Plant Material

A diverse set of field pea germplasm consisting of 171 genotypes was used for phenotypic and genotypic evaluation of B toxicity tolerance and PM resistance. An additional 22 genotypes were included in the salinity studies (Supplementary Table S1). Separate screening trials for B tolerance, salinity tolerance and PM resistance were conducted under controlled environment conditions at the Department of Economic Development, Jobs, Transport and Resources (DEDJTR), Horsham, VIC, Australia.

#### Boron Screening

Six seeds of each genotype were sown in 25 cm square non-draining pots filled with sandy loam soil pre-mixed with 10 mg kg^-1^ B as boric acid (H_3_BO_3_). Four genotypes were sown per pot and the seedlings were thinned to three per genotype 1 week post-emergence. Plants were watered on every third day to 100% field capacity after weighing the pots. The screening experiment contained three replications within a randomized complete block design and was conducted in a polyhouse maintained at 24 ± 4°C with a 16/8 h (light/dark) photoperiod.

Seven weeks after emergence, seedlings were assessed for B toxicity based on the percentage of leaf area affected per plant. A 1–6 scale was used where 1 had no leaf chlorosis or necrosis, 2 had ≤20% tip chlorosis and necrosis, 3 had 20–30% tip chlorosis and necrosis, 4 had 30–40% chlorosis and necrosis, 5 had 40–50% chlorosis and necrosis, and 6 had >50% chlorosis and necrosis of the total leaf area. A mean score was derived for all plants across all replications and genotypes were considered tolerant it they had a score of one or less or sensitive if the score was greater than 1.0.

#### PM Screening

Three seeds were sown in 14 cm diameter pots filled with Bio Gro^®^ potting mix in a glasshouse maintained at 20 ± 2°C, with a 16/8 h (light/dark) photoperiod. The potting mix consisted of 1,000 L of composted pine bark, 1 kg Floranid^®^ N32, 1 kg 8–9 months Osmocote^®^, 1 kg 3–4 months Osmocote^®^, 225 g MicroMax^®^ Complete, 225 g SP Quality^®^ FeEDDHA Chelate (6% Fe), 30 kg agricultural lime, and 2 kg Saturaid^®^. Following the method of Davidson et al. (2004), 2 weeks post emergence PM infected plants were distributed among the seedlings so that air-borne inoculum could spread around the trial by the glasshouse cooling fans. Seedlings were watered from below to prevent inoculum being washed off the leaves. The trial was arranged in three replicates in a randomized complete block design.

Seedlings were assessed for resistance 2 weeks after the introduction of PM infected plants and scored using a 0–4 scale, where 0 had no mycelial growth or sporulation, 1 had <5% mycelial growth and sporulation, 2 had 5–25% mycelial growth and sporulation, 3 had 25–75% mycelial growth and sporulation, and 4 had >75% mycelial growth and sporulation on the plant. Plant scores were averaged for each genotype and considered resistant if they had a mean score of less than or equal to one and were susceptible if they scored greater than one.

#### Salinity Screening

Salinity screening was conducted as described by Leonforte et al. (2013b). Briefly, six seeds of each genotype were sown 2 cm deep in 13 cm diameter pots into a 1:1 coarse river sand and 5 mm bluestone chip medium. Each pot was watered daily with rainwater until emergence. The seedlings were thinned to three per genotype 4 days post emergence and watered with Manutec hydroponic nutrient solution containing N (7.6%), P (3.1%), and K (18.2%) supplemented with 50 g L^-1^ calcium nitrate. After 10 days a salinity treatment mixed with the hydroponic nutrient solution was applied to all genotypes at an initial rate of 3 dS m^-1^ NaCl. Subsequently, every third day the concentration of NaCl was increased by 3 dS m^-1^ with each watering, up to 18 dS m^-1^, and maintained at this concentration until assessment. All solutions were applied at a rate of 300 ml per pot directly to the growing medium surface, avoiding contact with the leaves. The trial consisted of three replicates in a randomized complete block design. The salinity tolerance score and screening method were based on a visual growth response scale (1–10) as described by Leonforte et al. (2013a). The genotypes tested were then grouped into four response categories; tolerant (mean score ≤2.0), moderately tolerant (mean score >2 to ≤4.0), moderately sensitive (mean score >4.0 to ≤6.0) or sensitive (mean score >6.0).

#### Statistical Analysis

Analysis of variance (ANOVA) was conducted on all phenotyping data using GENSTAT 14th edition for windows.

### Genotyping

DNA was extracted from pooled leaf tissue samples harvested from up to three plants per genotype using the DNeasy^®^ 96 Plant Kit (QIAGEN, Hilden, Germany) according to the manufacturer’s instructions. The concentration of DNA was confirmed using a spectrophotometer (Thermo-Scientific, Wilmington, DE, USA) at the two wavelength ratios of A260/230 and A260/280 nm.

A near-diagnostic marker (PsMlo1) identified for PM that was also closely linked to B tolerance (Sudheesh et al., 2014) was assessed on a diverse set of pea germplasm. The sequence characterized amplified region (SCAR) primer pair; 5′-ATGGCTGAAGAGGGAGTT-3′ and 5′-GGTAGCAGCTTGATTTGTGGATA-3′ (Sudheesh et al., 2014) was synthesized and PCR amplifications were performed as described previously (Kaur et al., 2012). PCR products were combined with the ABI GeneScan LIZ500 size standard and analyzed using an ABI3730xl (Life Technologies Australia Pty Ltd, Scoresby, VIC, Australia) capillary electrophoresis platform according to the manufacturer’s instructions. Allele sizes were scored using GeneMapper^®^ 5 software package (Life Technologies Australia Pty Ltd). The PsMlo1 marker produced an allele size of 296 bp in B tolerant and PM resistant genotypes whereas the allele size of 294 bp was observed for sensitive/susceptible genotypes for both traits (Sudheesh et al., 2014).

SNP genotyping for the salt tolerance QTLs as described in Leonforte et al. (2013b) were performed using KASP^TM^ genotyping chemistry (LGC Genomics, Middlesex, UK) with some modifications. Primers were designed using 100 bp upstream and downstream flanking sequences around the target SNP variant position (Supplementary Table S2). The PCR reactions were carried out in 10 μl reaction volumes containing 50 ng genomic DNA, 5 μl KASP^TM^ 2x Master Mix (LGC Genomics) and 0.14 μl KASP^TM^ Primer Mix (LGC Genomics). The amplifications were performed in the SureCycler 8800 Thermal Cycler (Agilent Technologies, Inc., Santa Clara, CA, USA) using a touchdown PCR protocol following manufacturer’s instructions. End-point fluorescence values were determined by scanning the PCR plates in the FLUOstar Omega microplate reader (BMG LABTECH, Ortenberg, Germany) and the cluster plot analysis was completed in KlusterCaller^TM^ software (LGC Genomics).

## Results

### Phenotyping for Boron Tolerance and PM Resistance

**FIGURE 1:**
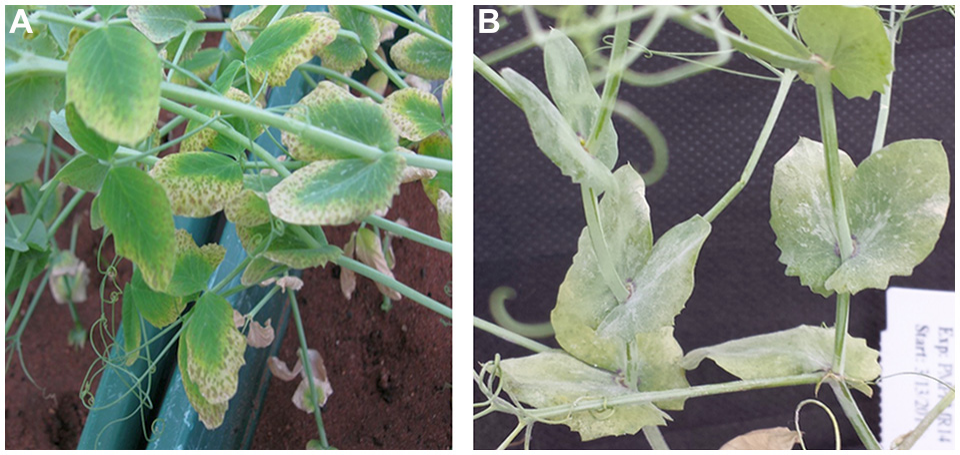
**Field pea symptoms of **(A)** B toxicity and **(B)** PM infection on leaf and stem**.

Plant responses within genotypes were generally consistent, although occasionally segregating. Individual genotypic responses under B treatment were uniform across the three replications and variance among the replications was non-significant (*P* = 0.259). However, the severity of B toxicity on genotypes varied significantly (*P* < 0.001) from 1 (no symptoms) to 6 (>50% chlorotic and necrotic). Of the 171 genotypes tested for B tolerance, 58 (34%) were tolerant and 113 (66%) were sensitive to B. B tolerant genotypes showed no symptoms of toxicity throughout the 7 weeks of the experiment. The sensitive genotypes showed chlorosis and brown speckles on older leaves as typical symptoms of B toxicity (**Figure 1A**).

Similar to B responses, plant responses to PM infection within genotypes were generally consistent, although occasionally segregating. Genotypes showed consistent response across the three replicates for PM disease (*P* = 0.762). The severity of PM infection varied significantly on genotypes (*P* < 0.001) from zero infection (no symptoms) to a highest mean score of 4 (>75% infection). Of the 171 genotypes tested for PM resistance, 61 genotypes (36%) were resistant and 110 (64%) were susceptible to PM disease with conidiospores present on both leaves and stems of the plants (**Figure 1B**).

**FIGURE 2:**
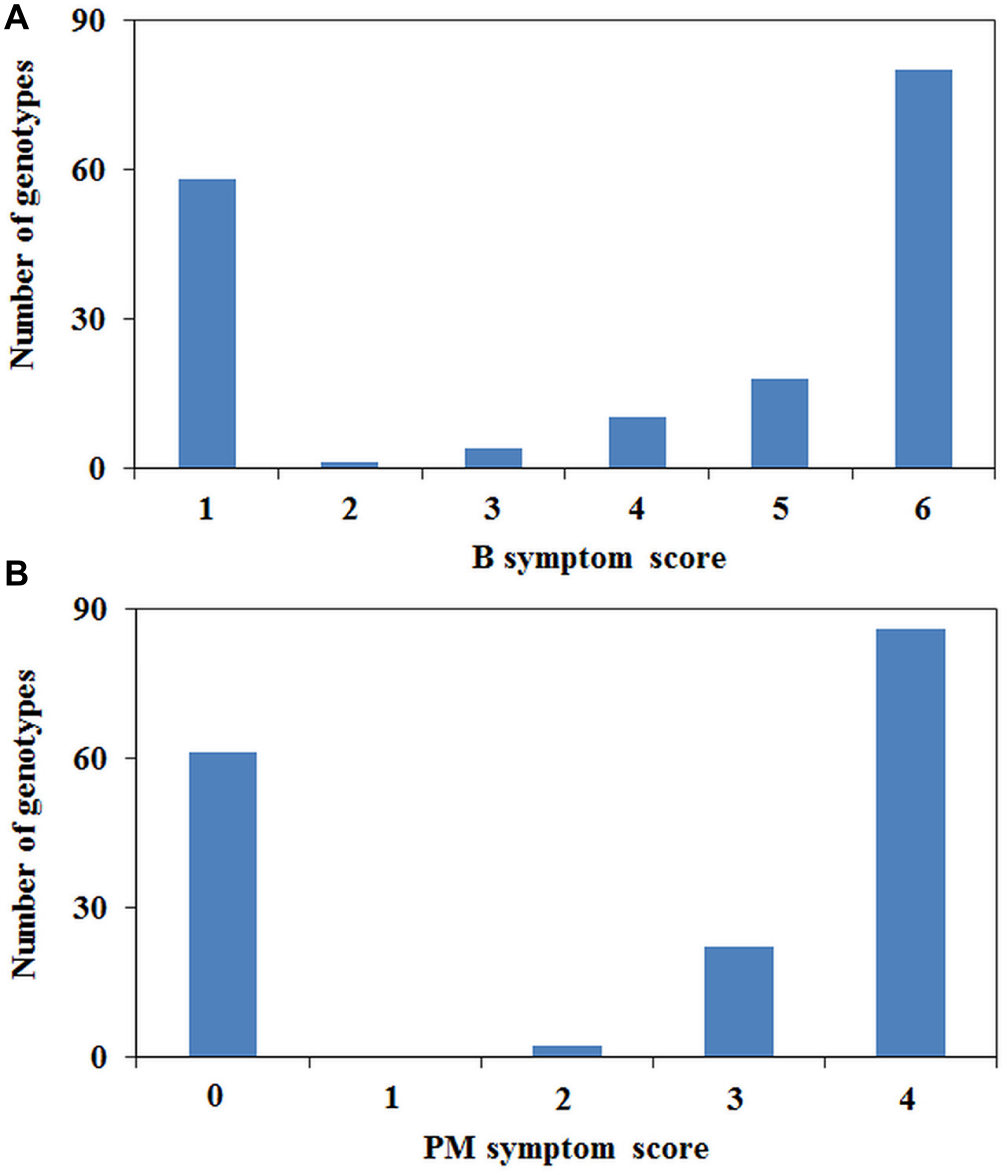
**Distribution of plant symptoms for **(A)** B and **(B)** PM phenotyping**.

The phenotypic scores between B tolerance and PM resistance were highly correlated (*r* = 0.959, *P* < 0.001). Fifty-six genotypes were tolerant to B and resistant to PM, and 108 genotypes were found to be sensitive/susceptible to both treatments. Of the 171 genotypes tested, only two genotypes (02-164-2 and 09HP300-10HO2-2) were tolerant to B but susceptible to PM, whereas five genotypes (05H097-06HOS2003, 05H347-06HOS2005, 06H310P-8, 06H362P-1, and 07H178P001) were sensitive to B but resistant to PM. B and PM phenotyping showed a bimodal distribution with the majority at the extremes of the scoring range, although there were a number of individuals with intermediate scores (**Figure 2**). The intermediate category contained a total of 24 genotypes for PM resistance and 33 for B tolerance of which 10 were common.

### Marker and Phenotype Correlation

The results for the marker analysis were highly correlated to the phenotypic results. Of the 171 genotypes tested, marker predictions were accurate for both traits for 135 genotypes (79%). A further seven genotypes were predicted accurately for PM, but not for B, generating 83% correct predictions for PM. These seven genotypes also showed an uncoupling of the two traits (**Table 1**).

**Table 1:** List of genotypes showing recombination between PM and B phenotypes and how they correlate with the PsMlo marker.

Genotypes	PsMlo1 allele^∗^	PM symptom score	B symptom score
**False positive (B)**			
05H097-06Hos2003	296	0 (R)	5 (S)^†^
05H347-06Hos2005	296	0 (R)	5 (S)^†^
06H310P-8	296	0 (R)	4 (S)^†^
06H362P-1	296	0 (R)	6 (S)
07H178P001	296	0 (R)	4 (S)^†^
**False negative (B)**			
02-164-2	294	4 (S)	1 (T)
09Hp300-10Ho2-2	294	4 (S)	1 (T)

*^∗^ “296” is the tolerant/resistant marker allele, “294” is the sensitive/susceptible marker allele, “S” is sensitive/susceptible, “T” is tolerant, “R” is resistant, and “^†^” represents the genotype segregated for the trait*.

A total of 26 genotypes were deemed false positives for both PM and B. These genotypes contained the resistance/tolerant allele but were susceptible/sensitive phenotypically. Furthermore, another three genotypes were false negatives for both traits, and contained the susceptible/sensitive marker allele and were phenotypically resistant/tolerant (**Table 2**). Of the PM false positives, two genotypes were segregating for the PM trait and 9 for B tolerance (**Tables 1** and **2**). The two genotypes segregating for PM resistance were also segregating for B tolerance. All segregating genotypes were identified as breeding lines. Of the 29 genotypes where the marker was not predictive, only 11 were publicly released cultivars. Six of the false positive genotypes were breeding lines with Parafield in the pedigree, which was also a false positive (**Table 2**).

**Table 2:** List of false positives and negatives genotypes of both traits with respect to the PsMlo marker.

Genotypes	PsMlo1 allele^∗^	PM symptom score	B symptom score
**False positive**			
Sydney	296	4 (S)	2 (S)^†^
00-254-28	296	4 (S)	6 (S)
01-226-2	296	3 (S)^†^	4 (S)^†^
05H245-06Hos2004	296	3 (S)	4 (S)^†^
06H461P-7	296	4 (S)	6 (S)
07H034P004	296	4 (S)	6 (S)
07H094P006	296	4 (S)	6 (S)
09Hp432-10Ho2-7	296	4 (S)	3 (S)^†^
95-072^∗^3	296	2 (S)^†^	3 (S)^†^
96-235^∗^5	296	4 (S)	6 (S)
97-031-6-3	296	4 (S)	6 (S)
97-724-5	296	4 (S)	6 (S)
Bonzer	296	4 (S)	5 (S)
Dunwa	296	4 (S)	6 (S)
Helena	296	4 (S)	6 (S)
Maro	296	4 (S)	6 (S)
Morgan	296	4 (S)	6 (S)
Odalette	296	4 (S)	6 (S)
P503-4-6-1	296	4 (S)	6 (S)
Parafield	296	3 (S)	6 (S)
Px-95-64-1-1	296	3 (S)	6 (S)
Px-96-79-8-1	296	4 (S)	6 (S)
Px-97-64	296	4 (S)	6 (S)
Santi	296	4 (S)	6 (S)
Soupa	296	4 (S)	6 (S)
Sturt	296	4 (S)	6 (S)
**False negative**			
04H341P-05Ho2010-1	294	0 (R)	1 (T)
09Hp288-10Ho2-7	294	0 (R)	1 (T)
09Hp375-10Ho2-3	294	0 (R)	1 (T)

*^∗^ “296” is tolerant/resistant marker allele, “294” is sensitive/susceptible marker allele, “S” is sensitive/susceptible, “T” is tolerant, “R” is resistant, and “^†^” represents the genotype segregating for the trait*.

### Salinity Phenotyping

**FIGURE 3:**
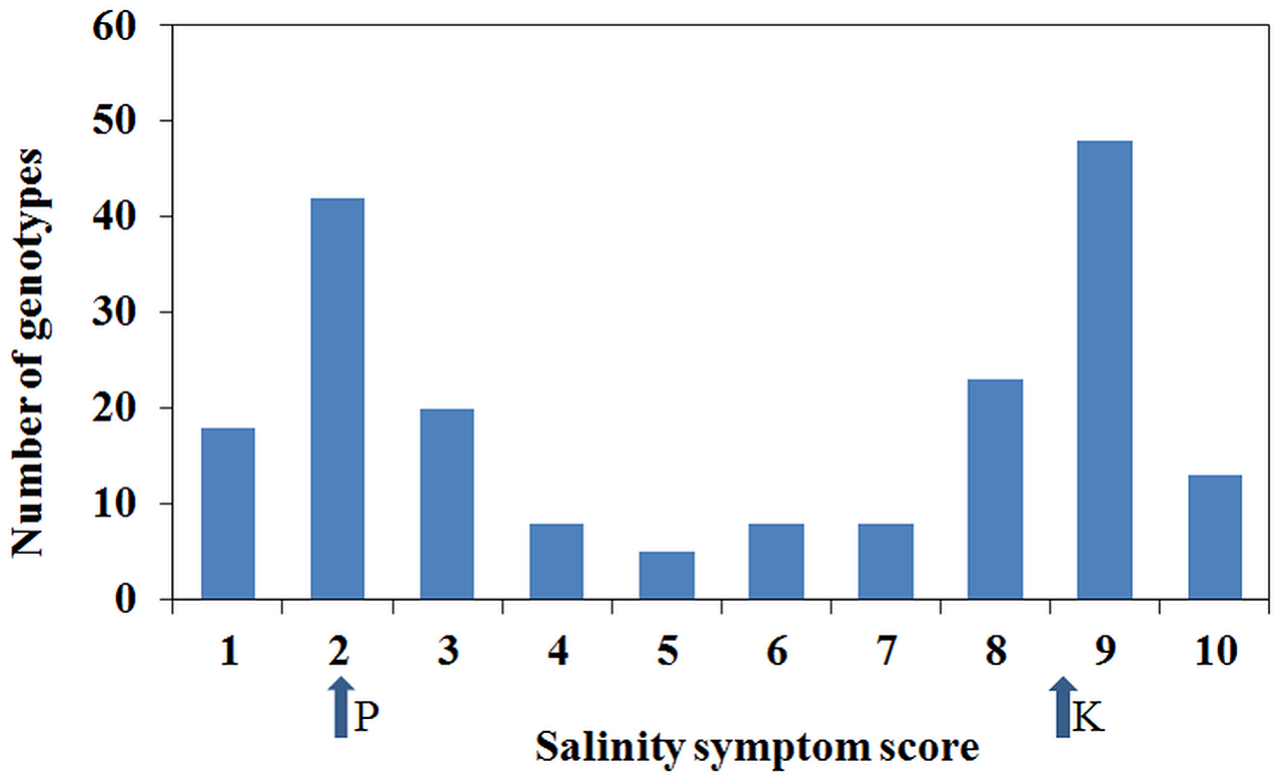
**Distribution of salinity symptoms scores from 1 to 10**. The arrows show the salinity scores for the parents, Parafield (P) and Kaspa (K), from the mapping population from which the salt QTLs were identified.

Plant responses within genotypes were generally consistent, although occasionally segregating. Individual genotypic responses in response to the salinity treatment were uniform across the three replications and variance among the replications was non-significant (*P* = 0.669). However, there was a significant (*P* < 0.001) variation among genotypes for salinity tolerance. Salinity phenotypes also showed a distribution with bimodal characteristics where the majority scored 2 or 9, although a number of individuals had intermediate scores (**Figure 3**). Based on the salinity symptom scores, genotypes such as Parafield, Yarrum, Collegian, and Dun showed tolerant responses with minor chlorosis on margins of lower leaves, whereas Kaspa, Maki and Bonzer were sensitive to the salinity treatment with severe chlorosis and necrosis on more than 50% of the plant.

### Salinity QTL Validation

The genotypes were grouped into four classes according to the alleles from the two loci of additive effect with a total Vp of 31%. Class 1 were the null genotypes where markers indicated that they had the sensitive alleles for both QTLs. This class had a total of 64 genotypes, of which 36 were sensitive or moderately sensitive, while 28 were tolerant or moderately tolerant to salinity. Class 2 had the tolerant allele from the Ps III QTL only and consisted of four genotypes that were phenotypically sensitive. Class 3 had the tolerant allele from Ps VII QTL, and consisted of 69 genotypes. Of these 69 genotypes, 30 were tolerant or moderately tolerant, 39 were sensitive or moderately sensitive. Class 4 had both tolerant QTL alleles and contained 56 genotypes. Of these, 28 were categorized as tolerant or moderately tolerant, while 28 were sensitive or moderately sensitive. Each class contained approximately 50% of genotypes as sensitive and moderately sensitive and 50% as tolerant or moderately tolerant, irrespective of the number of tolerance alleles derived from Parafield (**Table 3**). This indicates that the tolerance in Class 1 (and possibly Class 3) must be from different genetic sources.

**Table 3:** Number of genotypes with salinity symptom scores in four genotypic classes based on presence/absence of the salinity tolerance QTL markers.

Response categories	Class 1 (Null)	Class 2 (QTL 1)	Class 3 (QTL 2)	Class 4 (both QTLs)	Total
Sensitive	32	4	35	24	95
Moderately sensitive	4	–	4	4	12
Moderately tolerant	16	–	16	9	41
Tolerant	12	–	14	19	45
Total	64	4	69	56	193

**FIGURE 4:**
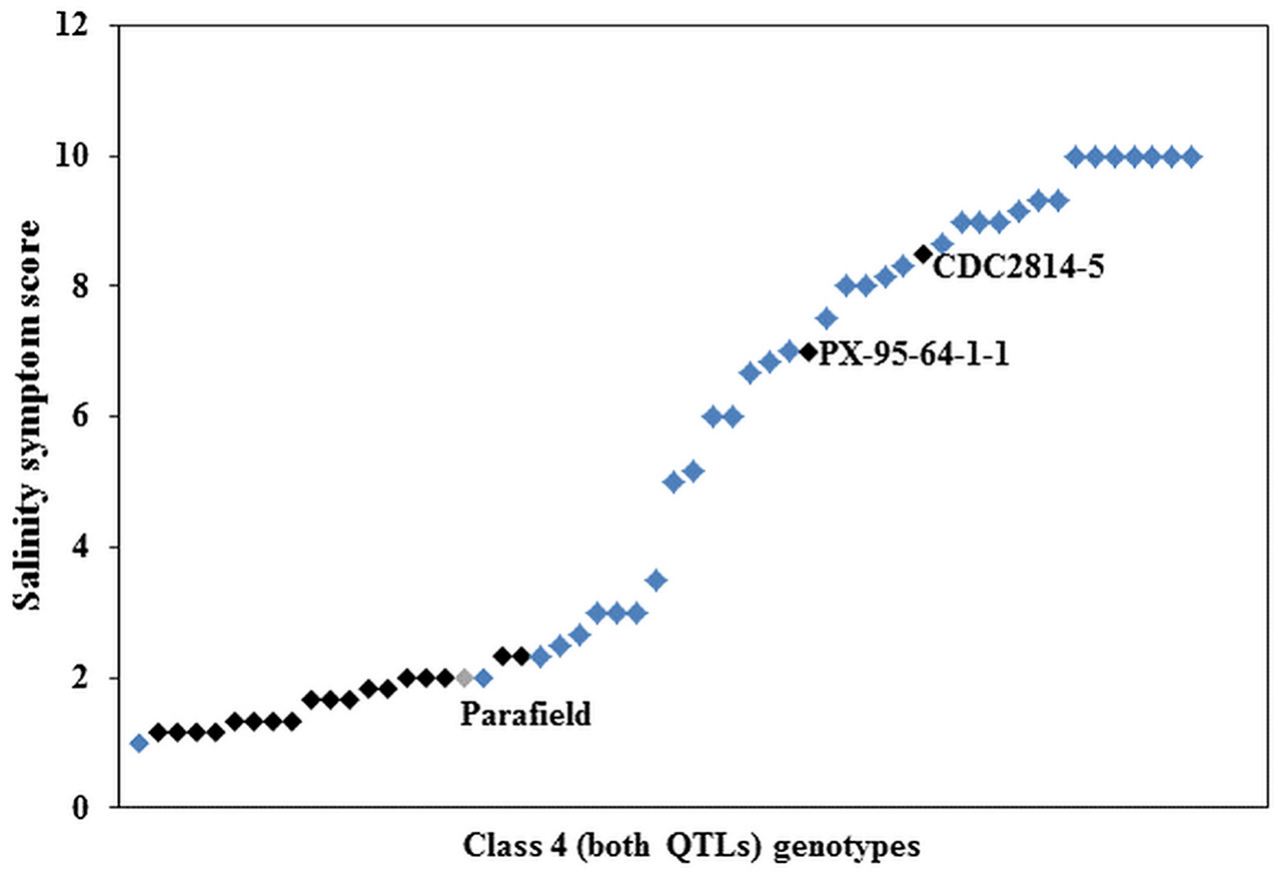
**Average salinity score of Class 4 genotypes, black squares (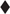) represent genotypes derived from the donor cultivar Parafield (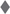) and blue squares (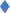) are non-Parafield derived genotypes**.

Further investigation of the genotypes within Class 4 showed that 21 had Parafield in their pedigree (Supplementary Table S3). Nineteen of the 21 genotypes were phenotypically tolerant, whereas two genotypes were sensitive to salinity (**Figure 4**). There were 35 genotypes of non-Parafield origin, and of these, only four (c. 11%) were tolerant to salinity, five (c. 14%) were moderately tolerant, four (c. 11%) were moderately sensitive, while the remaining 22 genotypes (c. 63%), were sensitive (**Figure 4**). Outside of Class 4 there were only three genotypes with Parafield in their pedigree and they belonged to Class 3, containing tolerant allele from Ps III QTL only.

## Discussion

The current study evaluates the application of the PsMlo1 marker and its predictive power for PM resistance and B tolerance as well as closely linked markers identified for salinity tolerance. The set of genotypes were identified to determine if the markers could be widely applied across diverse germplasm. The majority of the diverse germplasm set consisted of field pea breeding lines (82%) with the remainder cultivars. However, the selected set of genotypes served the objective of comparison between diagnostic and closely linked markers for breeding applications as the full range of genotypic and phenotypic classes were observed.

The PsMlo marker used in the current study is a SCAR marker that was developed from the genome sequencing of the full-length *PsMlo* gene in two resistant Australian field pea cultivars, Yarrum and ps1771 based on a 2-bp insertion in intron 11 (Sudheesh et al., 2014). The resistance to PM in field pea is known to be caused by loss of function at *Mlo* (Humphry et al., 2011; Pavan et al., 2011; Sudheesh et al., 2014). However, types of mutations in *Mlo* varies in different pea germplasm sets including frameshift mutations, insertions and premature termination of translation due to single base variations (Tiwari et al., 1997; Humphry et al., 2011; Pavan et al., 2011; Sudheesh et al., 2014). Co-mapping of PM resistance and B tolerance on Ps chromosome III suggests that both traits are controlled by major genes whose physical locations may be close to each other (Sudheesh et al., 2014). The phenotypic data from this study also showed strong linkage (*r* = 0.959) between PM resistance and B tolerance to confirm these findings. The frequency distribution of PM resistance and B tolerance showed a bimodal pattern in the diverse set of pea germplasm, with few genotypes in the intermediate classes which confirms that both traits are under simple genetic control. Only a few genotypes (4%) of the entire pea set showed intermediate responses for resistance/tolerance to PM and B indicating that other sources of partial resistance/tolerance could be present in the germplasm.

In most scenarios, the PsMlo1 marker correctly predicted the PM and B phenotypic responses (c. 84% for PM, c. 80% for B) across the pea germplasm set. However, a small proportion of genotypes were observed as false positives and false negatives for both traits. False positives are instances where the marker predicts the presence of the trait; however, this is not reflected in the phenotype. The most likely explanations for these inconsistencies would be a recombination event between the marker and the locus, or the presence of an alternative marker haplotype that is indistinguishable from the expected allele size. False negatives (when the marker predicts the absence of the trait but the phenotype is contradictory) are potentially more interesting as they may also represent a recombination event, or they may have a completely different source of resistance/tolerance available for exploitation by the breeding programs. The total number of false positives and negatives observed for B tolerance was higher compared to PM resistance which is expected as the marker was developed from the PM resistance gene, *PsMlo*, hence performed with better accuracy. A number of genotypes, especially breeding lines, were segregating for both traits where the marker should be used with more caution by genotyping and phenotyping single plants for marker confirmation. The high correlation of PsMlo marker to the phenotypes across the diverse set of pea germplasm, presumably indicates that locus *PsMlo1* (*er1*) is widespread within the gene pool. Two additional loci conferring PM resistance in field pea have been reported; *er2* locus on Ps III (Katoch et al., 2010) has effects largely confined to leaves (Marx, 1986; Tiwari et al., 1997) and *Er3* is a dominant gene introgressed into *P. pisi* from *P. fulvum* (Fondevilla et al., 2011). However, the possibility of *er2* and *Er3* genes being present in the current field pea diverse germplasm set is minimal (Antonio Leonforte pers comm), although their presence or other unknown genetic factors would explain false negative phenotypes.

The number of false positives was higher than the false negatives for both traits, which indicates that these genotypes may have common ancestory. Pedigree relationships of the field pea genotypes were investigated, and 16 of the 26 PM false positives had two common ancestors, Gottschalks Viktoria and breeder line 25/1-22-BI-1. These genotypes were used in the early 1980s, but are no longer available to test if one of these may be the source of the false positive haplotype. There were seven other false positives and six were introductions from the South Australian provincial breeding program. Their pedigree information only goes back to parents or grandparents; however, four of them have common ancestors of Alma and Wirrega. In the 1980s, the provincial breeding programs commonly swapped germplasm so it is possible that the false positive donor was exchanged between breeding programs, or there could have been multiple recombination events. Finally, there was one false positive, 96-235*5, that had a unique pedigree that was solely derived from the cultivars Morgan and Bohatyr. Pedigree information indicated that there were a limited number of germplasm donors that has given rise to the 26 PM false positive genotypes within the diverse set of pea germplasm.

In comparison to diagnostic markers for qualitative traits such as PM resistance and B tolerance, linked markers for quantitative traits such as salinity tolerance are much more challenging to validate across diverse germplasm. The markers for salinity tolerance used in the current study were developed from a mapping population of Kaspa × Parafield, in which Parafield, a moderately salt tolerant cultivar, contributed two QTLs explaining 12 and 19% of the phenotypic variation (Leonforte et al., 2013b). A low phenotypic variation contributed by the salinity QTLs reflects that these QTLs have minor effects. Similar observations have also been made by several other studies, where QTLs associated with various abiotic stresses including salinity have been shown to explain only a low percentage of phenotypic variation. This could either be due to environmental effects (Collins et al., 2008; Ashraf and Foolad, 2013) or epistatic interactions that can inhibit the expression of a favorable allele (Holland, 2001; Peleg et al., 2009).

The predictive power of the salinity tolerance markers was limited when tested across the diverse set of pea germplasm. This observation, however, is not surprising because these markers are associated with two QTLs of moderate effect in a bi-parental background and there could be greater potential for recombination. Furthermore, the effectiveness of these salinity markers was significantly improved for genotypes that have Parafield, the tolerance donor, in their pedigree. Within Class 4, 19 of the 21 Parafield derived genotypes (90%) were salt tolerant and had tolerant alleles from both QTLs. These results suggest that the markers have strong effects and worked reasonably well within the germplasm derived from Parafield. Similar responses of QTL linked markers have previously been shown in tomato where salinity tolerance QTLs worked well in offspring of populations from which they were originally developed (Monforte et al., 1997; Foolad, 2004; Ashraf and Foolad, 2013). Further examination of the Kaspa derived genotypes indicated that 76% of the sensitive genotypes had both sensitive marker alleles, whilst only 7% of the tolerant genotypes had both tolerant alleles, indicating that there were many false negatives. Over the last two decades, the field pea breeding program in Australia has moved away from Parafield type (fully leaved plants) to Kaspa type (semi-leafless plants), as the latter cultivar produced significant improvements in quality, agronomy and yield superiority. However, the breeding program has had a long history of selection under saline conditions with Parafield used in 39 crosses from 1995–2003, along with other salt donors. Kaspa came into the crossing program in 1997 and was used in 250 crosses up until 2007. Therefore, opportunities for Kaspa derived genotypes to pick up salt tolerance from other sources may have generated the high number of genotypes with tolerance. Given the quantitative nature of this trait, it is also possible that the diverse set of pea germplasm may contain many salt tolerance loci other than those contributed by Parafield.

## Conclusion

The PsMlo1 marker validation across diverse germplasm demonstrates that it can be used for selection for B tolerance and PM resistance in new germplasm sources different from the one in which the marker association was originally found. The PsMlo1 marker offers potential to be widely adapted to identify PM resistant and B tolerant germplasm, making this marker suitable for implementation within the pea breeding program. The fact that a single marker is associated with both traits is an added advantage. Our results demonstrate that markers linked to salinity tolerance QTLs can be implemented in germplasm derived from Parafield. The presence of other sources of tolerance to salinity suggest that there is potential to exploit other uncharacterized QTLs in the germplasm. Breeding for higher salinity tolerance therefore would require multiple QTLs for introgression into existing donor varieties in pea breeding programs. In view of the quantitative nature of salinity, the Parafield loci may not be sufficient for complete tolerance hence several mapping populations are currently being evaluated for new sources of tolerance to salinity. In addition, other genomic assisted approaches such as genome wide association studies (GWAS) and genomic selection (GS) may offer alternative solutions to understand polygenic traits with smaller genetic effects including salinity tolerance.

## Author Contributions

NC, AS, and SK contributed toward conception and interpretation of work. AL and PK organized peas germplasm for this work. SS and ML conducted genotyping work and data analysis. PK and MJ conducted phenotyping work and data analysis. GR, AS, SK and MJ drafted and critically revised the manuscript for important intellectual content and all authors approve final version of manuscript for publication.

## Conflict of Interest Statement

The authors declare that the research was conducted in the absence of any commercial or financial relationships that could be construed as a potential conflict of interest.

## ABBREVIATIONS

Fe-EDDHA: ferric ethylenediamine di-2-hydroxyphenyl acetate
GGP: great grandparent
GP: grandparent
MAS: marker assisted selection
P: parent
PM: powdery mildew
PsMlo1: mildew resistance locus O
QTL: quantitative trait loci
S: sensitive allele
T: tolerant allele
Vp: phenotypic variance
