# Supplementary material for: Validation of molecular markers associated with boron tolerance, powdery mildew resistance and salinity tolerance in field peas
Source: Front Plant Sci. 2015 Oct 27;6:917. doi: 10.3389/fpls.2015.00917 (PMC4621404; doi:10.3389/fpls.2015.00917)
Supplement: Supplementary file 1 [file Table_1.DOCX]

**Supplementary Table 1.** Genotypic data and phenotypic score of the field pea diversity set for the validation of PsMlo1 marker for boron (B) tolerance and powdery mildew (PM) resistance and two QTLs for salinity (SA) tolerance. R = resistant, S = sensitive and T = tolerant.

| Name | PsMlo1 | B score | PM score | SA score | QTL1 | QTL2 |
| --- | --- | --- | --- | --- | --- | --- |
| 00-254-28 | R | 6.0 | 3.5 | 2.0 | T | S |
| 00-254-32 | S | 6.0 | 4.0 | 1.8 | T | T |
| 00P040-2 |  |  |  | 1.7 | T | S |
| 00P040-5 | S | 5.9 | 4.0 | 8.7 | S | S |
| 01-226-2 | R | 4.1 | 2.5 | 9.0 | S | S |
| 01-228-2 | S | 5.7 | 4.0 | 8.7 | S | S |
| 01-230-27 | S | 6.0 | 4.0 | 3.0 | S | S |
| 01-230-33 | S | 6.0 | 4.0 | 5.2 | S | S |
| 01-230-5 | S | 6.0 | 4.0 | 4.3 | S | S |
| 01-256-14 | S | 6.0 | 4.0 | 3.0 | S | S |
| 01-269-6 | S | 5.2 | 4.0 | 1.3 | S | S |
| 01-284-2 | S | 6.0 | 4.0 |  |  |  |
| 01-290-9 |  |  |  | 8.3 | T | S |
| 01-303-3 | S | 6.0 | 4.0 | 8.7 | S | S |
| 01-503-7 | S | 6.0 | 4.0 | 7.3 | S | S |
| 01H280P-02HO2012-04HO5001 | S | 6.0 | 4.0 | 7.2 | T | S |
| 02-018-3 | S | 3.8 | 2.5 | 1.5 | T | S |
| 02-084-5 | S | 6.0 | 3.0 | 9.0 | S | S |
| 02-084-6 | S | 5.7 | 4.0 | 3.3 | T | S |
| 02-164-2 | S | 1.0 | 4.0 | 5.7 | T | S |
| 02-270-13 |  |  |  | 9.0 | S | S |
| 02-376-2 |  |  |  | 4.2 | S | S |
| 02-461-6 |  |  |  | 3.5 | T | T |
| 02-523-2 | R | 0.9 | 0.0 | 5.5 | T | S |
| 02-529-2 | R | 1.0 | 0.0 | 1.0 | T | T |
| 02-549-8 |  |  |  | 8.8 | T | S |
| 03-293-10-06HOS001 | R | 1.0 | 0.0 | 8.0 | T | S |
| 03H063-04HO2002-06NAS001 |  |  |  | 1.2 | T | T |
| 03H101P-04HO2003 | S | 6.0 | 4.0 | 2.3 | T | S |
| 03H142P-04H2006 |  |  |  | 2.8 | T | S |
| 03H160-04HO2001 | R | 1.0 | 0.0 |  |  |  |
| 03H173P-04H2006 | R | 1.0 | 0.0 | 3.0 | T | T |
| 03H211P-04HO2004 |  |  |  | 8.7 | T | S |
| 03H281P-04H2007 | R | 1.0 | 0.0 | 1.3 | T | T |
| 03H556P-04HO2024 | R | 1.0 | 0.0 | 1.7 | T | T |
| 04A145P-05HO2008 | S | 2.6 | 4.0 | 1.7 | T | S |
| 04H057P-05HO2003 | S | 6.0 | 2.0 | 5.7 | S | T |
| 04H150P-05HO2006 | S | 5.7 | 4.0 | 2.3 | S | S |
| 04H150P-05HO2007 | S | 5.3 | 3.0 | 2.2 | S | S |
| 04H306P-05HO2011 | R | 1.0 | 0.0 | 2.3 | S | S |
| 04H341P-05HO2010-1 | S | 1.0 | 0.0 | 1.3 | T | S |
| 04H341P-05HO2010-9 | S | 5.9 | 4.0 | 1.7 | S | S |
| 04H349P-05HO2005 | S | 5.7 | 4.0 | 8.0 | T | S |
| 04H448P-05HO2014 | R | 1.0 | 0.0 | 8.0 | S | S |
| 05H009-06HOS2005 | S | 6.0 | 4.0 | 9.0 | T | S |
| 05H097-06HOS2003 | R | 4.9 | 0.0 | 1.7 | S | S |
| 05H128-06TGVP002 | R | 1.0 | 0.0 | 6.3 | S | S |
| 05H141-06HOS2003 | S | 6.0 | 3.5 | 8.3 | S | S |
| 05H161P-1 | S | 5.8 | 4.0 | 9.0 | S | S |
| 05H245-06HOS2003 | S | 6.0 | 4.0 | 3.7 | S | S |
| 05H245-06HOS2004 | R | 4.3 | 3.0 | 8.3 | S | S |
| 05H247-06HOS2002 | S | 4.2 | 3.0 | 3.3 | S | S |
| 05H334-06HOS2003 | S | 5.2 | 4.0 |  |  |  |
| 05H346-06HOS2005 | S | 6.0 | 4.0 | 9.2 | S | S |
| 05H347-06HOS2005 | R | 5.1 | 0.0 | 8.0 | T | S |
| 05H371-06HOS2003 | S | 6.0 | 4.0 | 8.7 | S | S |
| 06H052P-2 |  |  |  | 3.2 | S | S |
| 06H061P-4 | S | 5.3 | 4.0 |  |  |  |
| 06H064P-3 | S | 5.1 | 3.0 | 9.3 | S | S |
| 06H093P-6 | S | 4.2 | 4.0 | 8.7 | S | S |
| 06H109P-9 | S | 6.0 | 4.0 | 2.7 | S | S |
| 06H213P-14 | S | 6.0 | 3.0 |  |  |  |
| 06H213P-6 | S | 6.0 | 3.0 | 7.3 | S | S |
| 06H246P-4 | S | 6.0 | 3.0 | 2.3 | T | S |
| 06H247P-8 |  |  |  | 2.0 | T | T |
| 06H254P-2 | S | 5.7 | 4.0 |  |  |  |
| 06H266P-7 | S | 6.0 | 3.0 | 8.7 | S | S |
| 06H310P-8 | R | 4.3 | 0.0 | 8.2 | S | S |
| 06H351P-1 | S | 6.0 | 3.0 | 3.3 | S | S |
| 06H357P-2 | S | 5.7 | 4.0 | 4.0 | S | S |
| 06H362P-1 | R | 6.0 | 0.0 | 7.7 | S | S |
| 06H364P-12 | S | 6.0 | 3.0 | 8.2 | S | T |
| 06H379P-6 | S | 5.7 | 3.0 | 1.3 | S | S |
| 06H392P-1 | S | 5.1 | 3.0 | 2.2 | T | S |
| 06H405P-3 | S | 6.0 | 4.0 |  |  |  |
| 06H408P-1 | S | 5.3 | 4.0 | 8.5 | T | S |
| 06H422P-3 | S | 5.3 | 4.0 |  |  |  |
| 06H428P-2 | S | 6.0 | 4.0 | 4.7 | S | S |
| 06H461P-7 | R | 6.0 | 4.0 | 3.7 | S | S |
| 07H015P005 | S | 5.1 | 4.0 | 3.3 | T | S |
| 07H033P006 | S | 4.9 | 4.0 | 2.0 | S | S |
| 07H034P002 | S | 5.7 | 4.0 | 1.5 | S | S |
| 07H034P004 | R | 6.0 | 4.0 | 8.7 | S | T |
| 07H034P008 | S | 5.4 | 3.0 |  |  |  |
| 07H036P003 | S | 5.7 | 4.0 | 1.8 | S | S |
| 07H036P007 | S | 5.4 | 4.0 | 8.7 | S | S |
| 07H094P002 |  |  |  | 2.2 | S | S |
| 07H094P006 | R | 5.9 | 4.0 | 2.3 | S | S |
| 07H098P004 | R | 1.0 | 0.0 | 2.0 | S | S |
| 07H099P004 | S | 4.3 | 4.0 |  |  |  |
| 07H102P003 |  |  |  | 1.3 | S | S |
| 07H105P003 |  |  |  | 8.3 | S | S |
| 07H111P001 | S | 6.0 | 4.0 | 2.7 | S | S |
| 07H115P004 | S | 3.2 | 4.0 | 8.0 | S | S |
| 07H116P006 | S | 5.7 | 4.0 | 1.7 | S | S |
| 07H142P007 | S | 6.0 | 3.0 | 8.5 | S | S |
| 07H144P006 | R | 1.0 | 0.0 | 9.2 | T | S |
| 07H144P008 | R | 1.0 | 0.0 |  |  |  |
| 07H147P003 | S | 5.2 | 4.0 | 8.3 | S | S |
| 07H178P001 | R | 4.2 | 0.0 | 1.8 | T | S |
| 07H190P004 |  |  |  | 4.0 | S | S |
| 07H194P002 | R | 1.0 | 0.0 | 1.7 | T | S |
| 07H203P001 |  |  |  | 6.8 | T | T |
| 07H208P007 | R | 1.0 | 0.0 | 1.7 | S | S |
| 07H210P001 | R | 1.0 | 0.0 | 2.2 | T | S |
| 07H210P002 | R | 1.0 | 0.0 | 1.3 | T | S |
| 07H218P001 | R | 1.0 | 0.0 | 4.0 | T | S |
| 07H226P001 |  |  |  | 1.0 | T | S |
| 07H226P006 |  |  |  | 1.3 | T | S |
| 07H226P007 | R | 1.0 | 0.0 | 1.3 | T | T |
| 07H259P007 | R | 1.0 | 0.0 |  |  |  |
| 09HP278-10HO2-3 | R | 1.0 | 0.0 | 8.2 | T | S |
| 09HP283-10HO2-12 | S | 3.7 | 4.0 | 9.0 | T | T |
| 09HP287-10HO2-1 | R | 1.0 | 0.0 | 3.0 | T | S |
| 09HP287-10HO2-3 | R | 1.0 | 0.0 | 8.3 | S | T |
| 09HP288-10HO2-1 | S | 6.0 | 3.0 | 2.2 | T | S |
| 09HP288-10HO2-7 | S | 1.0 | 0.0 | 8.8 | T | S |
| 09HP289-10HO2-7 | R | 1.0 | 0.0 | 8.5 | T | T |
| 09HP300-10HO2-2 | S | 1.0 | 4.0 | 5.2 | T | T |
| 09HP340-10HO2-4 | R | 1.0 | 0.0 | 7.5 | T | T |
| 09HP375-10HO2-3 | S | 1.0 | 0.0 | 9.0 | T | S |
| 09HP380-10HO2-1 | R | 1.0 | 0.0 | 8.2 | T | T |
| 09HP382-10HO2-3 | R | 1.0 | 0.0 | 8.7 | T | S |
| 09HP382-10HO2-6 | R | 1.0 | 0.0 | 8.7 | T | S |
| 09HP432-10HO2-7 | R | 3.2 | 4.0 | 2.2 | T | S |
| 2000-1532 |  |  |  | 9.0 | T | S |
| 86-55P*32-11 | S | 4.0 | 3.0 | 7.0 | T | T |
| 88-011P9*9-4 |  |  |  | 8.0 | T | T |
| 89-003P9*9-5-3 |  |  |  | 6.0 | T | T |
| 89-036P8*3-1-1 |  |  |  | 9.7 | T | S |
| 89-116P8*16-1 |  |  |  | 1.3 | T | T |
| 90-027P8*32-5 |  |  |  | 1.2 | T | T |
| 91-025*50-1 |  |  |  | 3.0 | T | S |
| 92-190P5*6 | S | 5.5 | 4.0 | 8.0 | S | S |
| 92-208*12 | S | 5.2 | 4.0 |  |  |  |
| 92-218*8-5 | R | 1.0 | 0.0 | 9.0 | T | T |
| 93-062*14 | S | 5.5 | 4.0 | 1.8 | T | S |
| 94-208*4 | S | 5.5 | 4.0 | 7.2 | T | S |
| 95-072*3 | R | 3.2 | 2.0 | 2.3 | T | T |
| 96-049*8 |  |  |  | 3.0 | T | T |
| 96-120*2 | R | 1.0 | 0.0 | 8.5 | T | S |
| 96-120*4 | R | 1.0 | 0.0 | 9.0 | T | S |
| 96-151*1 |  |  |  | 1.3 | T | T |
| 96-235*5 | R | 6.0 | 4.0 | 3.0 | T | S |
| 96-262*3 | R | 1.0 | 0.0 | 9.0 | T | S |
| 96-286*1-11 | S | 6.0 | 4.0 | 2.3 | T | S |
| 96-286*1-16 | S | 6.0 | 4.0 | 4.5 | T | S |
| 96-288*1 | S | 5.7 | 4.0 | 10.0 | T | T |
| 97-031-6-3 | R | 6.0 | 4.0 | 9.0 | S | S |
| 97-031-6-6 | S | 6.0 | 4.0 | 9.0 | S | S |
| 97-033*4 | S | 6.0 | 4.0 | 6.3 | S | S |
| 97-724-5 | R | 6.0 | 4.0 | 2.7 | T | S |
| ALMA |  |  |  | 2.3 | T | T |
| BIGDADDY | S | 6.0 | 4.0 | 6.0 | T | S |
| BLUEY |  |  |  | 2.5 | T | T |
| BOHATYR |  |  |  | 9.7 | T | S |
| BONZER | R | 5.4 | 4.0 | 10.0 | T | S |
| BUNDI | S | 5.7 | 4.0 | 1.3 | S | S |
| CDC Striker | S | 6.0 | 3.0 | 10.0 | T | S |
| CDC2814-5 | R | 1.0 | 0.0 | 8.3 | T | T |
| CDC2819-12 | R | 1.0 | 0.0 | 10.0 | T | T |
| CDC2936-7 | R | 1.0 | 0.0 | 9.3 | T | S |
| CDC2942-4 | R | 1.0 | 0.0 | 2.0 | T | S |
| CDC2949-19 | R | 1.0 | 0.0 |  |  |  |
| CDC2949-20 | R | 1.0 | 0.0 | 8.7 | T | T |
| CDC2949-7 | R | 1.0 | 0.0 | 9.2 | T | S |
| CDC3007-6 | R | 1.0 | 0.0 | 10.0 | T | T |
| CDC3034-16 | R | 1.0 | 0.0 | 10.0 | T | T |
| CDC3094-5 | R | 1.0 | 0.0 | 10.0 | T | T |
| CDC3094-7 | R | 1.0 | 0.0 | 10.0 | T | T |
| CDC3100-4 | R | 1.0 | 0.0 | 9.3 | T | T |
| CDC3100-5 | R | 1.0 | 0.0 | 6.7 | T | T |
| CDC3151-6 | R | 1.0 | 0.0 | 9.0 | T | T |
| CDC3159-11 |  |  |  | 9.3 | T | S |
| CDC3168-9 | R | 1.0 | 0.0 | 10.0 | T | S |
| CDC3519-8 | R | 1.0 | 0.0 |  |  |  |
| COLLEGIAN |  |  |  | 1.2 | T | T |
| COOKE | S | 5.8 | 4.0 | 9.3 | T | T |
| CRESSY-EARLY BLUE |  |  |  | 3.0 | T | T |
| DINKUM |  |  |  | 9.2 | T | T |
| DUN |  |  |  | 1.8 | T | T |
| DUNDALE (TRCI) |  |  |  | 2.7 | T | T |
| DUNWA | R | 6.0 | 4.0 | 2.0 | T | T |
| EIFFEL | S | 6.0 | 3.5 | 3.0 | T | S |
| EXCELL |  |  |  | 8.7 | T | S |
| GLENROY |  |  |  | 2.0 | T | T |
| HELENA | R | 5.7 | 4.0 | 1.7 | T | T |
| HIGHLIGHT | R | 1.0 | 0.0 | 10.0 | T | T |
| JUPITER |  |  |  | 1.0 | T | S |
| KASPA | S | 5.9 | 4.0 | 8.7 | S | S |
| MAITLAND |  |  |  | 2.3 | T | T |
| MAKI | R | 1.0 | 0.0 | 9.0 | T | S |
| MARO | R | 6.0 | 4.0 |  |  |  |
| MOONLIGHT | S | 6.0 | 4.0 | 9.0 | T | S |
| MORGAN | R | 6.0 | 4.0 |  |  |  |
| MUKTA | R | 1.0 | 0.0 | 8.3 | T | S |
| ODALETTE | R | 6.0 | 4.0 | 5.0 | T | T |
| OZP1102 |  |  |  | 2.0 | T | T |
| P503-4-6-1 | R | 5.9 | 4.0 |  |  |  |
| PARAFIELD | R | 6.0 | 3.0 | 2.0 | T | T |
| PARAVIC | S | 6.0 | 4.0 |  |  |  |
| PSL4-RRSEL-1 | S | 6.0 | 4.0 | 8.7 | S | S |
| PX-95-64-1-1 | R | 5.7 | 3.0 | 7.0 | T | T |
| PX-96-57-8 |  |  |  | 1.7 | T | T |
| PX-96-59-1-2 | R | 1.0 | 0.0 |  |  |  |
| PX-96-79-8-1 | R | 5.7 | 4.0 | 1.5 | T | S |
| PX-97-64 | R | 5.9 | 4.0 | 9.0 | T | S |
| SANTI | R | 5.9 | 4.0 |  |  |  |
| SNOWPEAK |  |  |  | 9.0 | S | S |
| SOUPA | R | 6.0 | 4.0 | 10.0 | T | S |
| STURT | R | 6.0 | 4.0 | 6.0 | T | T |
| SUPRA | S | 5.7 | 4.0 |  |  |  |
| Sydney | R | 2.3 | 4.0 | 8.0 | T | S |
| WIRREGA |  |  |  | 8.0 | T | T |
| YARRUM | R | 1.0 | 0.0 | 1.2 | T | T |
