# Supplementary material for: Validation of molecular markers associated with boron tolerance, powdery mildew resistance and salinity tolerance in field peas
Source: Front Plant Sci. 2015 Oct 27;6:917. doi: 10.3389/fpls.2015.00917 (PMC4621404; doi:10.3389/fpls.2015.00917)
Supplement: Supplementary file 2 [file Table_2.DOCX]

**Supplementary Table 2**. Flanking marker sequences used for salinity QTL’s.

| SNP_100000318 | GTCACCTTGCTAAGTCTAGGCATTTGACGAAGCAATCTCTGAGTTGGATACTCTGAG[T/C]GAGGAGTCTTACAAAGACAGCACATTGATTATGCAGCTTCTGAGGGACAACCTCACATTGTGGACTTCAGACATCCCTGAAGAAGGAGTTGAGGAACAAA |
| --- | --- |
| SNP_100000130 | TTATGAGTATATAGAAGAAGATATCTTCAACAAAAGCAATCTCCTATTGGAATTGAACCCGGTTCATAAGAAAATTCCGGTTCTTGTTCATGGCCAGAAA[T/C]CAATAGCAGAGTCACTTATTATCCTTGAATACATTGATGAAACATGGAAACAATATCCATTGTTGTCTCCCCATCCTTATCAAAGAGCTCTTGCTCGGTT |
| SNP_100000313 | GCTAAAAGGGTTTCATTTCATATATAATTATGTCACTGGTTGAAGGTAGCAATGAAACCAATAGGTCCTTTTCCAGAAACAGCAGCTTGAATTGCAAAGA[T/C]AAGGAAAACCACCATGGCCAAGCGAGAATGCTTAATCTCTGCCAGTTGAAGTCTTTCTTTCTCTTCAGGATCATTGGCCAAACCAAGAGGATCAAAGAAC |
| SNP_100000353 | TGACGTGCATTCGTCCTCGTCGTCCTAATAGCAATAAAACAGAGCAAGTTCTCAGTTGCCATGTCCTGTTAGTGAGAAGCTCATGACTGTTTGAGCTATG[T/G]GTTGCACATTTTCTTCCTCTATGATAAATGTGTTTTGAAGTTTAAATATATCTGAGGTAGATGTATAAAGTAGGAAGCACATTACCTGAGGATGGTGCCA |
