# Supplementary material for: Validation of molecular markers associated with boron tolerance, powdery mildew resistance and salinity tolerance in field peas
Source: Front Plant Sci. 2015 Oct 27;6:917. doi: 10.3389/fpls.2015.00917 (PMC4621404; doi:10.3389/fpls.2015.00917)
Supplement: Supplementary file 3 [file Table_3.DOCX]

**Supplementary** **Table 3** Parafield derived germplasm from the field pea diversity set and their corresponding salinity score and QTL status

| Genotypes | Parafield relationship† | Salinity symptom score | Ps III†† | Ps VII†† |
| --- | --- | --- | --- | --- |
| Parafield | L | 2.0 | T | T |
| 96-151*1 | P | 1.3 | T | T |
| PX-96-57-8 | P | 1.7 | T | T |
| 03H556P-04HO2024 | GP | 1.7 | T | T |
| DUN | GGP | 1.8 | T | T |
| 89-116P8*16-1 | GGP | 1.3 | T | T |
| Collegian | GGP | 1.2 | T | T |
| 00-254-32 | GGP | 1.8 | T | T |
| Glenroy | GGP | 2.0 | T | T |
| OZP1102 | GGP | 2.0 | T | T |
| 90-027P8*32-5 | GGP | 1.2 | T | T |
| 03H281P-04H2007 | GGP | 1.3 | T | T |
| Dunwa | GGP | 2.0 | T | T |
| Helena | GGP | 1.7 | T | T |
| Yarrum | GGP | 1.2 | T | T |
| 03H173P-04H2006 | GGP | 3.0 | T | T |
| 95-072*3 | GGP | 2.3 | T | T |
| Alma | GGP | 2.3 | T | T |
| Maitland | GGP | 2.3 | T | T |
| PX-96-79-8-1 | GGP | 1.5 | S | T |
| CDC2814-5 | GGP | 8.3 | T | T |
| PX-95-64-1-1 | GGP | 7.0 | T | T |
| PX-97-64 | GGP | 9.0 | S | T |
| 2000-1532 | GGP | 9.0 | S | T |

† “L” represent the line, Parafield, “P” represents that Parafield was a parent, GP a grandparent and GPP a great grandparent.

†† “T” represents the tolerant allele from the marker and “S” the sensitive allele.
